# Impact of work aspects on communication, emotional intelligence and empathy in nursing^*^

**DOI:** 10.1590/1518-8345.2933.3118

**Published:** 2019-01-31

**Authors:** María del Carmen Giménez-Espert, Vicente Javier Prado-Gascó, Selene Valero-Moreno

**Affiliations:** 1 Universidad de Valencia, Facultad de Enfermería y Podología, Valencia, Comunidad Valenciana, Spain.; 2 Universidad de Valencia, Facultad de Psicología, Valencia, Comunidad Valenciana, Spain.

**Keywords:** Communication, Emotional Intelligence, Empathy, Nursing, Work Satisfaction, Emotional Abilities, Comunicação, Inteligência Emocional, Empatia, Enfermagem, Satisfação no Trabalho, Habilidades Emocionais, Comunicación, Inteligencia Emocional, Empatía, Enfermería, Satisfacción Laboral, Habilidades Emocionales

## Abstract

**Objective:**

to assess the impact of the type of contract and seniority of nursing staff on their attitudes towards communication, emotional intelligence and empathy.

**Methods:**

the instruments measuring attitudes towards communication, empathy and emotional intelligence. The study sample was composed of 450 nurses from 7 hospitals. To explore the effect of the variables studied, one-factor ANOVA test, Pearson correlations and hierarchical multiple linear regression models were performed according to the type of contract.

**Results:**

there are statistically significant differences between the variables studied according to the type of contract. More specifically, the nursing staff with permanent contract showed higher scores in the cognitive dimension of the scale attitudes towards communication. Likewise, the highest correlations were found among the dimensions of attitudes towards communication in all the groups. At the same time, seniority was positively related to emotional intelligence in the interim and negatively related to empathy in the permanent contract. Finally, regarding the regression models, it was observed that the perspective taken is the main predictor of each dimension of attitudes towards communication in all the groups, regardless of the type of contract.

**Conclusion:**

positive working conditions (job security, permanent contract and seniority) have been found to influence the communication skills in the patient-nurse relationship.

## RESUMO

**Objetivo:**

determinar o impacto do tipo de contrato e do tempo de serviço prestado da equipe de enfermagem nas suas atitudes em relação à comunicação, inteligência emocional e empatia.

**Métodos:**

os instrumentos que avaliam as atitudes em relação à comunicação, empatia e inteligência emocional. A amostra do estudo consistiu de 450 enfermeiras de 7 hospitais. Para avaliar o efeito das variáveis estudadas, foram realizados ANOVA unilateral, correlações de Pearson e modelos de regressão linear hierárquica múltipla, de acordo com o tipo de contrato.

**Resultados:**

há diferenças estatisticamente significativas entre as variáveis estudadas de acordo com o tipo de contrato. Mais especificamente, a equipe de enfermagem com contrato permanente apresentou pontuações mais elevadas na dimensão cognitiva da escala atitudes em relação à comunicação. Do mesmo modo, as maiores correlações foram encontradas entre as dimensões da escala atitudes em relação à comunicação, em todos os grupos. Ao mesmo tempo, o tempo de serviço foi positivamente relacionado à inteligência emocional no contrato provisório, e negativamente com a empatia no contrato permanente. Por fim, observando os modelos de regressão, observou-se que a tomada de perspectiva é a principal preditora em cada dimensão da escala atitudes em relação à comunicação em todos os grupos, independentemente do tipo de contrato.

**Conclusão:**

comprovou-se que condições de trabalho positivas (segurança no trabalho, contrato permanente e tempo de serviço prestado) influenciam a capacidade de comunicação na relação paciente-enfermeira.

## RESUMEN

**Objetivo:**

determinar el impacto del tipo de contrato y la antigüedad del personal de enfermería en sus actitudes hacia la comunicación, la inteligencia emocional y la empatía.

**Métodos:**

los instrumentos que miden las actitudes hacia la comunicación, la empatía y la inteligencia emocional. La muestra del estudio fue de 450 enfermeras de 7 hospitales. Para comprobar el efecto de las variables estudiadas, se realizaron ANOVA de un factor, correlaciones Pearson y modelos de regresión lineal jerárquica múltiple, según el tipo de contrato.

**Resultados:**

existen diferencias estadísticamente significativas entre las variables estudiadas según el tipo de contrato. Más concretamente, el personal de enfermería con contrato indefinido mostró puntuaciones más altas en la dimensión cognitiva de la escala actitudes hacia la comunicación. Asimismo, las mayores correlaciones se encontraron entre las dimensiones de la escala actitudes hacia la comunicación en todos los grupos. Al mismo tiempo, la antigüedad se relacionaba positivamente con la inteligencia emocional en el ínterin y negativamente con la empatía en el contrato indefinido. Finalmente, mirando los modelos de regresión, se observó que la toma de perspectiva es el principal predictor de cada dimensión de la escala actitudes hacia la comunicación en todos los grupos, independientemente del tipo de contrato.

**Conclusión:**

se ha comprobado que las condiciones de trabajo positivas (seguridad laboral, contrato permanente y antigüedad) influyen en la capacidad de comunicación en la relación paciente-enfermera.

## Introduction

Interpersonal communication between nurse practitioner and patient is an essential part of health care^1^. The ability to communicate helps in the adherence to treatment and can increase the effectiveness of treatments in primary care^2^. It can also contribute to reducing the emotional distress associated with different diseases^3^, increase disease control, reduce anxiety, improve treatment follow-up, generate realistic expectations, contribute to patient safety, promote self-care and participation^4^, and even prevent malpractice complaints and suits^5^. Despite the importance of the quality of communication with patient, few studies have explored this subject in depth. Previous reports in the literature have approached this subject considering communication in a general way, but without studying the relation between communication and the specific characteristics of the patient or disease^6^. Communication can be influenced by the knowledge and attitudes of the nurses themselves^7^, the emotional intelligence (EI) and the empathy of the professionals^8^. These variables may act as barriers and/or facilitators, promoting or hindering effective communication with the patient^9^. Overall, studies suggest that nurses who lack EI and empathy do not have the ability to communicate effectively neither with the patient nor with their team^10^ ^-^ ^11^. 

Few studies have addressed the relationship between attitudes towards communication, EI and empathy^12^ ^-^ ^13^. These variables could be influenced by personal or socio-demographic^14^ characteristics or conditions of employment, such as seniority or type of contract^15^. In this sense, uncertainty about working conditions^16^, as well as lack of experience of nursing professionals, may increase the levels of stress and tension in the workplace, which could directly affect the communication style of nursing professionals^17^ ^-^ ^18^, as well as their empathy and EI. Despite the importance of all these variables, no previous study aiming at analyzing the impact that the working conditions may have on these constructs or how they could relate to each other was found. Therefore, the central objective of this study was to analyze the role that the type of contract and seniority may have in the attitudes towards communication, empathy and EI, as well as in the relationships between these variables in nursing professionals. For this reason, the following hypotheses were raised: H1: People with high level of seniority will exhibit an increased emotional competence; H2: High levels of EI and empathy (see from patient’s point of view) will be positively related to the affective dimensions, negatively related to Compassionate Care and Perspective-Taking (JSE); H3: Permanent contracts will give the nurses greater peace of mind and satisfaction, which in turn will modulate the relationship between the remaining variables of the study.

## Method

The study was composed of 450 nurses who perform direct patient care from 7 public hospitals in Valencia. After obtaining authorization from the nursing supervisors, an informed consent was obtained from the participants. The nurses filled-out the questionnaires (35 minutes in length) and placed them in the polling places located in the different units; after two weeks, reminder messages were sent and after 3-4 weeks, the questionnaires were collected from the polling places. Of the 1,124 questionnaires distributed, 460 were collected: 10 were eliminated because they were over 40% left blank. The response rate was 40.93% and the error rate was 4.6%. The collection phase was from June 2015 to March 2016. It is a study with a cross-sectional design within a single temporal moment.

The instruments and variables used in this study were: 

Questionnaire on nurses’ attitudes towards communication (ACO)^11^ ^-^ ^12^. It is composed of 25 items grouped into three dimensions to evaluate the attitudes towards communication: affective, conative and cognitive. This instrument has shown adequate psychometric properties: Satorra-bentler scaled chi-square test (S-B χ^2^); Degrees of freedom (df) S-B χ^2^ (df)=525.09 (272); χ^2^(df)=4.90; Root Mean Square Error of Approximation (RMSEA); Confident Interval (CI); RMSEA (CI)=0.045 (0.037-0.057); Comparative Fit Index (CFI), CFI=0.91, Non-Normed Fit Index (NNFI), NNFI=0.90, IFI=0.91; Affective: Confidence Interval for Cronbach’s Alpha (CIα)=0.95 (0.94-0.96); Composite reliability coefficient (CRC), CFC=0.95; Average variance extracted (AVE), AVE=0.60; Conative: CIα=0.92 (0.90-0.93), CRC=0.91, AVE=0.53; Cognitive: CIα=0.85 (0.82-0.87), CRC=0.85, AVE=0.58^11^.

Jefferson Scale of Empathy in Nursing Students adapted from Jefferson Scale of Physician Empathy (JSPE)^19^ and translated by the research team. Jefferson Scale of Physician Empathy was adapted for nursing students in its original version^19^ and it consists of 19 items (JSE) grouped into three factors to assess empathy: perspective-taking, compassionate care and thinking like the patient. It presents adequate psychometric properties: S-B χ^2^(df)=174.74 (87); χ^2^(df)=3.82; RMSEA (CI)=0.047 (0.037-0.057); CFI=0.92, NNFI=0.90, IFI=0.91; Perspective-taking: Confidence Interval of Cronbach’s Alpha (CIα)=0.87 (0.85-0.89), CRC=0.88, AVE=0.47; Compassionate care: CIα=0.78 (0.75-0.81), CRC=0.78, AVE=0.48; Standing in patients’ shoes: CIα=0.76 (0.71-0.80), CRC=0.76, AVE=0.61^13^.

Trait Meta-Mood Scale (TMMS24). This scale presents 24 items grouped in three dimensions: emotional attention, emotional clarity and emotional repair. The Spanish version, translated by Fernández-Berrocal and adapted to the nursing context^20^ allows us to evaluate EI. It presents adequate psychometric properties in nurses’ population: S-B χ^2^(df)=370.20 (149); χ^2^ (df)=3.58; RMSEA (CI)=0.057 (0.050-0.065); CFI=0.91, NNFI=0.90, IFI=0.91; Emotional attention: Confidence Interval of Cronbach’s Alpha (CIα)=0.80 (0.77-0.83), CRC=0.80, AVE=0.45; Emotional Clarity: CIα=0.87 (0.85-0.89), CRC=0087, AVE=0.46; Emotional Repair: CIα=0.85 (0.82-0.87), CRC=0.85, AVE=0.49^21^.

The analysis of data was as follows. First, the differences in the variables under study were analyzed by means of one-factor ANOVA, according to the type of contract. Afterwards, Pearson correlations between the variables under study and seniority were calculated according to the type of employment contract. Finally, hierarchical multiple linear regression models were analyzed taking into consideration the type of contract. 

This study was approved by the Research Ethics Committee of the University of Valencia, under protocol H1432032268924, and the Clinical Research Ethics Committees (CEIC) of the selected hospitals. All participants gave their consent to participate.

## Results

This study was composed of 450 nurses who perform direct patient care from seven public hospitals in Valencia, whose ages varied from 22 to 64 years, and with an average age of 44.13 (SD=11.58). In terms of gender, 75.6% were women (313) and 24.4% were men (101). Regarding the employment situation of participants, 53.8% (239) were permanent position, compared to 28.4% (126) who were temporary workers and 17.8% (79) who were interim employees. The seniority or care experience ranged from 5 months to 43 years and 3 months, with an average of 18 years and 3 months (M=218.49; SD=148.89 (months), and a median of 5 years and 3 months.

Regarding the differences in ACO, JSE and TMMS according to the type of contract, statistically significant differences were found (*p*≤0.05) only in the case of the cognitive dimension (F_2_=3.52; p=0.03; η^2^=0.02) of the ACO scale. The Tukey *post-hoc* test indicated the existence of differences between the following groups (*p*=0.04): nursing staff with permanent employment contracts showed a slightly higher score than the interim staff (I-J=-0.27) (Table 1).

**Table 1:** Dimensions of the Attitudes towards Communication, Jefferson Scale of Empathy and Trait Meta-Mood Scale, according to the type of contract. Valencia, Spain, 2015-2016

	**Dimension**	**Temporary contract**		**Interim contract**		**Permanent contract**		F*	**p** ^**†**^	η^**2‡**^
		**M** ^**§**^	**DT** ^**|||**^	**M** ^**§**^	**DT** ^**|||**^	**M** ^**§**^	**DT** ^**|||**^			
**ACO** ^**¶**^	**Affective**	**1,61**	**0,86**	**1,62**	**0,98**	**1,55**	**0,84**	**0,30**	**0,74**	**-**
	**Conative**	**4,10**	**0,82**	**4,18**	**0,88**	**4,29**	**0,76**	**1,73**	**0,18**	**-**
	**Cognitive**	**4,40**	**0,86**	**4,38**	**0,98**	**4,61**	**0,69**	**3,52**	**0,03**	**0,02**
**JSES**	**Perspective-Taking**	**4,54**	**0,54**	**4,52**	**0,60**	**4,52**	**0,57**	**0,03**	**0,97**	**-**
	**Compassionate Care**	**1,79**	**0,84**	**1,92**	**0,95**	**1,88**	**0,88**	**0,48**	**0,62**	**-**
	**Standing in patient’s shoes**	**1,98**	**1,01**	**2,07**	**1,09**	**1,88**	**0,88**	**0,13**	**0,88**	**-**
**TSMM24** ^**††**^	**Emotional Attention**	**3,64**	**0,77**	**3,46**	**0,82**	**3,62**	**0,74**	**1,86**	**0,16**	**-**
	**Emotional clarity**	**3,86**	**0,55**	**3,81**	**0,71**	**3,83**	**0,72**	**0,13**	**0,88**	**-**
	**Emotional repair**	**3,80**	**0,70**	**3,83**	**0,84**	**3,80**	**0,74**	**0,07**	**0,93**	**-**

*F: F-test value to perform one-factor ANOVA; ^†^ *p*: level of significance; ^‡^η^2^: partial eta squared; ^§^M: mean; ||SD: standard deviation; ^¶^ACO: Attitudes towards Communication; **JSE: Jefferson Scale of Empathy; ^††^TSMM24: Trait Meta-Mood Scale-24.

Regarding the correlations between ACO, JSE and TMMS24 according to the type of contract, the next step was to analyze the relationship between the study variables and age, both for the total sample and for the type of contract. In all cases, statistically significant correlations were found between most of the dimensions of the three scales. In the three groups, the highest correlations were observed between the dimensions of each scale separately. No statistically significant correlation was observed between the dimensions of the ACO scale and the emotional attention dimensions of the TMMS24 in any of the groups. As regards seniority, it was not significantly related (*p*≥0.05) to any of the dimensions in the case of the temporary work contract. However, a positive and moderate relationship between emotional care (TMMS24) and seniority (r=0.59, *p*≤0.01) was found for nursing staff with a permanent position. In addition, in the case of interim employees, seniority was significantly associated, in a moderately and negatively way, with standing in patients’ shoes (JSE) (r=-0.44, *p*≤0.01). 

**Figure 1:**
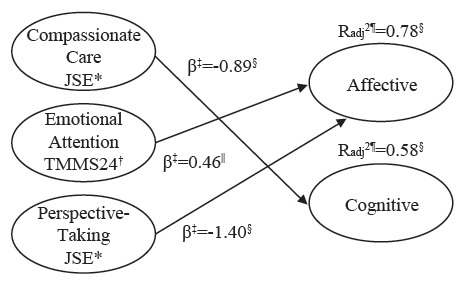
Prediction of ACO (Attitudes towards Communication) by EI (Emotional Intelligence) and Empathy, with model according to temporary contracts, Valencia, Spain, 2015-2016

**Figure 2:**
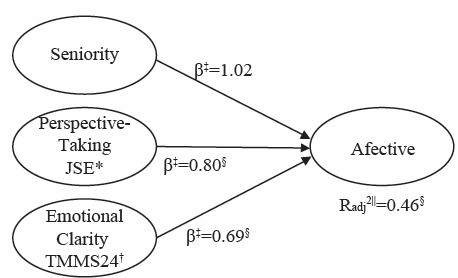
Prediction of ACO (Attitudes towards Communication) by EI (Emotional Intelligence) and Empathy, with model according to interim positions, Valencia, Spain, 2015-2016

**Figure 3:**
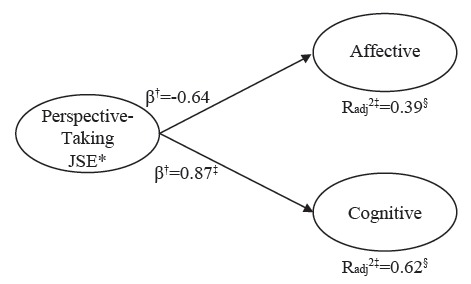
Prediction of ACO (Attitudes towards Communication) by EI (Emotional Intelligence) and Empathy, with model according to permanent positions, Valencia, Spain, 2015-2016

Finally, the predictive power of the variables under study was analyzed by means of a hierarchical regression according to the type of contract with the ACO dimensions as criterion variables. In the first step, seniority was included; in the second step, all dimensions of the JSE questionnaire were added; and in the last step, TMMS24 variables were also included. Seniority could not significantly predict any of the dimensions of ACO, regardless of the type of contract. When including the dimensions of the JSE scale, it was possible to predict 78% of the variance in the affective dimension (*p*≤0.001) (Figure 1), 58% in the cognitive dimension (*p*≤0.01) and 35% in the conative dimension (*p*=0.07) (Figure 1), although it was not significant in the case of the temporary contract. In the case of interim employees (Figure 2), the dimensions of the JSE could not significantly predict any of the dimensions of the ACO. Finally, in the sample of nursing professionals with permanent contract (Figure 3), the dimensions of the JSE scale could predict 24% of the affective variance (*p*=0.02), 61% of the conative variance (*p*≤0.001) and 62% of the cognitive variance (*p*≤0.001). 

*JSE: Jefferson Scale of Empathy; ^†^TMMS24: Trait Meta-Mood Scale-24; ^‡^β: standardized beta coefficient; ^§^: level of significance *p*≤0.01; ^||^: level of significance *p*≤0.05; ^¶^ Radj^2^: Adjusted R-squared.

*JSE: Jefferson Scale of Empathy; ^†^TMMS24: Trait Meta-Mood Scale-24; ^‡^β: standardized beta coefficient; ^§^: level of significance *p*≤0.01; ^||^: Radj^2^: Adjusted R-squared; ^¶^: level of significance *p*≤0.05;

*JSE: Jefferson Scale of Empathy; ^†^β: standardized beta coefficient; Radj^2‡^= Adjusted R-squared; ^§^: level of significance *p*≤0.05

The inclusion of the TMMS24 dimensions in the third and final step did not significantly improve the explanatory capacity of the model in the case of interim workers, but rather in the case of temporary and permanent employment contracts. Figure 1 shows the relationship between the variables of the study according to the type of contract in this last step. Only those variables found to be significant predictors have been included in the figure.

## Discussion 

Communication, EI and empathy are necessary skills in nursing care^5^ ^,^ ^12^ ^,^ ^16^. Different variables can influence these skills, such as working conditions for example. However, there are no studies analyzing these variables. Therefore, one of the objectives of this study was to determine the impact of labor issues (type of contract and seniority) of nursing professionals in their attitudes toward communication, IE and empathy.

Despite the contributions of the present research, it presents some limitations: the sampling procedures are not probabilistic and are not representative of all nursing professionals, which makes it difficult to generalize the results found.

The results of the present study partially support hypothesis 1, in which a greater seniority is associated with a greater emotional competence. This association is only observed in emotional attention and standing in patients’ shoes, as indicated in previous studies^2^. Likewise, hypothesis 2 is also partially supported by our results, since the affective dimension (ACO) is negatively related with perspective-taking (ACO), and positively with standing in patients’ shoes, but compassionate care is not negatively related, as expected. On the other hand, the dimension perspective-taking (JSE) is the variable that best predicts the affective and cognitive dimensions of communication, regardless the type of contract (position). In the regression, nurses with an interim position show a negative beta coefficient with respect to the affective dimension, which differs from the rest of the groups. Considering that this variable is measured in a reverse order (higher scores indicate worse empathic capacity), this result can be explained if we consider that this type of contract implies a greater workload and uncertainty about the contract length, which can generate stress and affect the type of attitude towards communication^17^ ^-^ ^18^. Finally, regarding the predictive models of ACO, JSE and TMMS24, according to seniority, EI and empathy have a greater predictive power in the case of temporary contracts than in the rest of the groups. These results do not seem to be in line with hypothesis 1, in which it is suggested that a permanent contract improves the satisfaction of nurses and modulates their emotional skills, as indicated in previous studies^15^ ^-^ ^16^. Perhaps these results can be explained by the fact that people with a permanent contract are usually older people, which in essence could suggest, as reported in the literature, that young people have greater emotional ability, both because of their attitude and the current type of training, which gives priority to these aspects^5^. The lack of emotional skills training courses also shows that nursing professionals with a permanent contract present better cognitive skills with respect to attitudes towards communication, according to one-factor ANOVA. The reason for this is that most nurses have a permanent contract and a smaller percentage of nurses are hired on a temporary basis, which is not proportionate.

One of the main limitations related to the methodology used in this study is that the regression models allow establishing associations between the variables studied. However, they do not allow establishing causal associations, so it was not possible to generalize that working conditions were responsible for the emotional skills, but simply that they are related. By its nature, linear regression is based only on linear relationships between dependent and independent variables. That is, it is assumed that there is a straight-line relationship between them. Another limitation of the methodology used is that the means of the analyzed variables are used in the regression. Just as the mean is not a complete description of a single variable, linear regression is not a complete description of the relationships between variables. Along with this limitation there is another possible bias characteristic of the regression, the outliers, although these outliers have been eliminated before performing the analysis, in order to decrease their influence on the relationships analyzed. In this case, it can be said that this bias has been controlled to prevent it from influencing the results. 

These limitations or biases related to the methodology used will be taken into account in future studies. This type of methodology, although simple, is one of the main ones used in the different disciplines, such as nursing and psychology. Therefore, this study is particularly interesting due to the paucity in the literature of studies that have analyzed the importance of emotional skills in nurses’ attitudes toward communication, while also considering the impact of work aspects on such relationships. It would be interesting to carry out studies aiming at analyzing in depth this relationship, although it is not possible to demonstrate the impact of work aspects on emotional skills, but the results obtained need to be considered with caution.

## Conclusion 

This study shows how work aspects seem to influence the attitudes towards communication, EI and empathy, as well as the relationships established between them. It has been also found that positive work aspects (job security, permanent contract and seniority) influence the number of emotional skills in the patient-nurse relationship.
